# Developing Item Banks for Measuring Pediatric Generic Health-Related Quality of Life: An Application of the International Classification of Functioning, Disability and Health for Children and Youth and Item Response Theory

**DOI:** 10.1371/journal.pone.0107771

**Published:** 2014-09-30

**Authors:** Pranav K. Gandhi, Lindsay A. Thompson, Sanjeev Y. Tuli, Dennis A. Revicki, Elizabeth Shenkman, I-Chan Huang

**Affiliations:** 1 Department of Pharmacy Practice, School of Pharmacy, South College, Knoxville, TN, United States of America; 2 Department of Pediatrics, College of Medicine, University of Florida, Gainesville, FL, United States of America; 3 Outcomes Research, Evidera, Bethesda, MD, United States of America; 4 Department of Health Outcomes and Policy, College of Medicine, University of Florida, Gainesville, FL, United States of America; 5 Department of Epidemiology and Cancer Control, St. Jude Children's Research Hospital, Memphis, TN, United States of America; TNO Quality of Life, Netherlands

## Abstract

The purpose of this study was to develop item banks by linking items from three pediatric health-related quality of life (HRQoL) instruments using a mixed methodology. Secondary data were collected from 469 parents of children aged 8-16 years. The International Classification of Functioning, Disability and Health-Children and Youth (ICF-CY) served as a framework to compare the concepts of items from three HRQoL instruments. The structural validity of the individual domains was examined using confirmatory factor analyses. Samejima's Graded Response Model was used to calibrate items from different instruments. The known-groups validity of each domain was examined using the status of children with special health care needs (CSHCN). Concepts represented by the items in the three instruments were linked to 24 different second-level categories of the ICF-CY. Eight item banks representing eight unidimensional domains were created based on the linkage of the concepts measured by the items of the three instruments to the ICF-CY. The HRQoL results of CSHCN in seven out of eight domains (except personality) were significantly lower compared with children without special health care needs (*p*<0.05). This study demonstrates a useful approach to compare the item concepts from the three instruments and to generate item banks for a pediatric population.

## Introduction

Over the past decade, pediatric research has shifted its attention from advancing treatments and survival rates for children with various diseases and disorders to improving their functional status and health-related quality of life (HRQoL). In parallel to this paradigm shift, the World Health Organization (WHO) elaborated the concept of health by emphasizing its components and determinants, which include body functions, body structures, activities and participation, and environmental and personal factors [1], [2]. The use of this bio-psycho-social model helps us understand the psychological, social, and environmental determinants of health outcomes, especially in children and adolescents with various diseases, at different developmental stages and who are often under-served [3]–[5].

Several generic- and disease-specific HRQoL instruments have been developed for use in pediatric populations. The commonly used generic HRQoL instruments are the Child Health and Illness Profile (CHIP) [6], the Child Health Questionnaire (CHQ) [7], the KIDSCREEN-52 [8], the KINDL-R [9], and the Pediatric Quality of Life Inventory (PedsQL) [10]. Ideally, pediatric HRQoL instruments should be brief in content, related to the child's age and developmental stage, and demonstrate good measurement properties such as reliability, validity, and responsiveness [11]. In addition, good generic instruments should be able to assess the HRQoL in children with varying health conditions and across different languages and cultures [12], [13]. Using a large sample of children enrolled in the Medicaid program, our previous study based on the classical test theory (CTT) method suggested that none of the existing pediatric HRQoL instruments (the CHIP, KIDSCREEN, KINDL-R, or PedsQL) was superior to any other in the different psychometric properties [14], [15]. Although the CTT has often been applied to compare or develop new HRQoL instruments, the use of the CTT alone may neglect item-level information, which might bias the conclusions in comparative effectiveness research and clinical applications [16]. Using qualitative methodologies to compare the heterogeneity of item content, followed by advanced quantitative methodologies (e.g., item response theory; IRT) [16], [17] to quantify the measurement properties of individual items for the design of appropriate pediatric instruments, is important [4], [18].

The International Classification of Functioning, Disability and Health for Children and Youth (ICF-CY) taxonomy represents an international classification system for pediatric health and disability. The ICF-CY has been used in research and clinical practice to understand the components and determinants of pediatric health outcomes and to help design HRQoL instruments [19]. The ICF-CY can also serve as a framework to compare the contents of items in existing HRQoL instruments, thus providing evidence of content validity [5], [20], [21]. Although researchers have used the ICF-CY to investigate the contents of items in pediatric HRQoL instruments [4], [5], [22]–[24], few studies have used the ICF-CY to conduct head-to-head comparisons among items from different pediatric HRQoL instruments combined with quantitative methods to validate these items and generate item banks for use in pediatric settings [25].

Designing and administering HRQOL items in children must account for several pediatric issues related to age, neurocognitive development, and special health care needs. Although there is a consensus that children as young as 8 years old are able to and should self-report their HRQoL [26], [27], the use of parent-proxy reports remains important, especially when the children are mentally disabled, too young, or too sick to self-report [26], [28], [29]. Parent-proxy reports of a child's HRQoL is of particular importance for children enrolled in public insurance programs such as Medicaid [30] because the parents are responsible for evaluating their child's health outcomes and making decisions for health services. Children from low-income families and those enrolled in Medicaid have a greater likelihood of a poorer HRQoL related to multiple chronic conditions than children of high-income families and those in private health insurance programs [31].

The main purpose of this study was to use a mixed qualitative and quantitative methodology to develop initial items banks on the basis of three most frequently used pediatric legacy HRQoL instruments (KIDSCREEN-52, KINDL-R, and PedsQL). The KIDSCREEN-52 and KINDL-R are among the most widely used pediatric generic HRQoL instruments in Europe, whereas the PedsQL is the most popular generic instrument in the United States. These three instruments capture the common aspects of HRQoL including physical, psychological and social domains, and are suitable for children and adolescents. Our first objective was to apply the ICF-CY framework to compare the contents of items from three pediatric legacy instruments and map these items into the domains suggested by the ICF-CY. The second objective was to apply IRT methodology to develop item banks using items mapped to the same HRQoL domain represented by the ICF-CY. The advantage of the IRT is that it can calibrate items from different instruments on the same metric by capturing comparable underlying constructs of HRQoL. The developed item banks were further validated using the Children with Special Health Care Needs (CSHCNs) Screener [32].

## Methods

### Study sample, inclusion and exclusion criteria, and data collection

The secondary data were analyzed using a 2009 survey comprising parents who had children aged 2–17 years old who were enrolled in Florida's Medicaid program. Only families with 12 months of continuous enrollment in Medicaid were considered eligible for this study. Eligible families were sent a primer letter followed by a phone call for recruitment (n = 5,789). Families with disconnected and non-working numbers were excluded (n = 2,783). A total of 908 parents among the remaining 3,006 eligible participants completed the telephone interview (response rate 30.2%: 908/3006) by trained interviewers using a structured questionnaire including three legacy instruments (KIDSCREEN-52, KINDL-R, and PedsQL) and demographic information. Age-appropriate versions of the instruments were administered to each child and adolescent (Table 1). The present study analyzed the data of 469 surveys that were collected from the parents of children aged 8–16 years to allow for the comparison of all items across the three instruments.

**Table 1:** Descriptions of the three pediatric health-related quality of life (HRQoL) instruments used in this study.

	KIDSCREEN-52	KINDL-R	PedsQL
Number of items	52	24	23
Number of subscales/domains	10	6	4
Age ranges suggested by the original instrument	8–18 years old	Kiddy KINDL: 4–7	Toddler: 2–4
		Kid KINDL: 8–12	Young child: 5–7
		Kiddo KINDL: 13–16	Child: 8–12
			Teen: 13–18
Age range used in this study	8–16 years old	8–16 years old	8–16 years old
Item scoring	5-point Likert scale	5-point Likert scale	5-point Likert scale
	Items scored so higher scores indicate better HRQoL	All items scored so that higher scores indicate better HRQoL	All items reverse scored so higher scores indicate better HRQoL

The University of Florida's Institutional Review Board approved the study protocol. This study is part of the Quality Assurance project of the Florida Health Department for public insurance programs, and the survey was conducted via telephone. Per the University's IRB, we obtained a waiver for collecting written informed consent. Instead, we collected verbal agreement from all study participants over the phone when they were enrolled.

### Survey instruments

The three pediatric legacy instruments, the KIDSCREEN-52, KINDL-R, and PedsQL, were chosen for the present study and are given in Table 1. Additionally, the parent participants answered the CSHCN Screener [32] for the analysis of the known-groups validity of the item banks. The domains and total scores were transformed into a 0–100 point scale (100 =  best HRQoL and 0 =  worst HRQoL). The characteristics of the three HRQoL instruments and the CSHCN Screener are described as follows:

The KIDSCREEN-52 is the most commonly administered pediatric HRQoL instrument in Europe [8], [33]. The instrument contains 10 domains (52 items) including physical well-being (5 items), psychological well-being (6 items), moods and emotions (7 items), self-perception (5 items), autonomy (5 items), parent relationship and home life (6 items), social support and peers (6 items), social acceptance and bullying (3 items), school environment (6 items), and financial resources (3 items).

The KINDL-R was developed to assess HRQoL in healthy, chronically ill, and acutely ill children [9]. The Kid/Kiddo KINDL was especially designed for older children and adolescents between 8 and 16 years of age. Each version has 6 domains with 4 items per domain. The domains include physical well-being, psychological well-being, self-esteem, family functioning, friends, and school functioning.

The PedsQL 4.0 was developed to assess the WHO's core concept of health (physical, mental, and social functioning) plus school functioning [10]. This instrument contains 23 items measuring the problems associated with performing daily functions. The four domains include physical functioning (8 items), emotional functioning (5 items), social functioning (5 items), and school functioning (5 items).

CSHCN are defined as having a chronic condition (physical, developmental, behavioral, or emotional) and requiring health-related services and/or medication. The CSHCN Screener consists of five questions to evaluate the presence and duration of health conditions captured by three domains (dependency on prescription medications, service use above routine levels, and functional limitations). If a parent responds “yes” to a health consequence item, two follow-up items are asked to determine if the consequence is due to a medical or health condition. Both follow-up items must be answered “yes” to qualify the child as a CSHCN for that domain.

### Mapping, linking, and validation methodology

#### The ICF-CY framework

This study used the ICF-CY as a framework to link the items from the three pediatric HRQoL instruments. The ICF-CY includes four major components, and each has its respective classification codes and categories. The four components are ‘body functions’, represented as code letter b; ‘body structure’, represented as code letter s; ‘activities and participation’, represented as code letter d; and ‘environmental factors’, represented as code letter e. The numeric codes following these letters represent the chapter number (one digit), the second level (two digits), and the third and fourth levels (one digit each). The letters with the suffix of numeric codes are termed as categories. In this study, the items from the three HRQoL instruments were linked to the second-level categories of the ICF-CY [18], and these categories were used to form the domains of HRQoL and the item banks. The specific steps to map, link, and calibrate the items from the three instruments and to develop and validate the item banks are described as follows:

#### Step 1: Mapping items from the three pediatric HRQoL instruments using the ICF-CY framework

The rules suggested by Cieza et al. [18] were used to link the meaningful concepts of the items in the three HRQoL instruments to the ICF-CY. Prior to linking the items, the meaningful concepts of individual items were extracted by two authors using a data extraction form (see below). Per Cieza et al. [18], when the concept from each item of the three HRQoL instruments could not be linked with a specific ICF-CY category, the item was identified as ‘not definable (nd)’ [18]. For example, the KINDL item of ‘my child worried about his/her future’ was assigned ‘nd’ because it could not be represented by any specific ICF-CY category. Additionally, the abbreviation ‘nc’ (not covered) was used when the ICF-CY classification did not include a specific concept of the item [18].

In addition to the rules of Cieza et al. [18], other rules for item linkage were applied in this study. If an item from the three HRQoL instruments was linked to more than one category of the ICF-CY, multiple categories were reported for that particular item. However, to create different item banks with each measuring a unidimensional concept of HRQoL, we chose the most relevant ICF-CY category to represent the content of each individual item. In line with the rules of Cieza et al. [18], the linking procedure was conducted independently by two authors (PG and ICH). To resolve any disagreements, a third rater was consulted, and a final decision based on a consensus among the three raters was made. Items that were linked from different instruments and placed in the same ICF-CY category are supposed to measure the same underlying construct of HRQoL. Subsequently, specific domains were created for these items that capture the same underlying constructs, and individual item banks were developed to represent the specific domains of HRQoL.

#### Step 2: Psychometric analysis using IRT

Confirmatory factor analyses (CFA) were used to test the structural validity of the individual domains. Specifically, CFA was used to test unidimensionality and local independence, which are two basic assumptions of IRT analysis. For the unidimensionality of individual domains, various fit indices were used to assess the structural validity, including the goodness-of-fit index χ^2^ (a non-significant chi-square indicates a good model fit) and the root mean square error of approximation (RMSEA) (a value below 0.08 indicates a good model fit, and values below 0.05 indicate a close fit) [34]. Items with acceptable magnitudes of factor loading on the corresponding domains (λ>0.4; *p*<0.05) were considered to be appropriate for the IRT application. Non-significant items and items with a lower factor loading (λ<0.4; *p*>0.05) were removed from the analysis. For local independence, residual correlations of paired items from the same domains were investigated, and one of the paired items with a high residual covariance (>10.0) was considered for removal because both items might measure similar content.

Following the CFA, we used Samejima's Graded Response Model (GRM), a unidimensional IRT model for items with categorical response categories, to test and calibrate item parameter estimates and to calculate domain scores for specific HRQoL domains that were identified in the previous step. We examined different measurement properties to further remove some items from the item banks, including item thresholds and discrimination as well as item and test information functioning (IIF/TIF). Items with discrimination values>1.0, higher or lower threshold values (i.e., able to measure easiest or most challenging underlying HRQoL, respectively), and a higher IIF were considered for retention. Additionally, standardized local dependence (LD) χ^2^ statistics were examined to test local independence between paired items in a specific domain [35]. Items that failed to satisfy more than one of the criteria described above were deleted from the item banks. Various fit indices were adopted to assess the appropriateness of individual domains, including marginal reliability estimates (≥0.60 as acceptable), the *M*_2_ statistic [36], [37], and RMSEA (<0.08 as a good model fit for unidimensionality and <0.05 as a close fit).

#### Step 3: Validation analysis using the known-groups approach

The CSHCN Screener [32] was used to investigate the known-groups validity of item banks measuring different domains of pediatric HRQoL. Known-groups validity was demonstrated when the mean HRQoL domain scores derived from the item banks were able to better discriminate among the clinically meaningful groups (i.e., CSHCN versus children without special health care needs). For each individual, the underlying HRQoL domain scores were estimated using an expected a posteriori estimation (EAP) based on the Bayesian statistical principle. Bivariate relationships were examined using an independent t-test to compare the mean difference in the underlying HRQoL domain scores between the two groups. Additional analyses comparing the underlying HRQoL scores between the two groups were conducted with adjustments for the children's age and gender and for the parent's race and education. Cohen's effect size (ES; Cohen's d) estimates were reported to indicate the magnitude of difference in the domain scores between CSHCN and children without special health care needs divided by the smaller value of the standard deviation (SD). Cohen's d values of <0.2, 0.2–0.49, 0.5–0.79, and>0.8 were regarded as a negligible, small, moderate, and large ES, respectively [38].

LISREL 8.8 was used to perform CFA [39], the Item Response Theory for Patient-Reported Outcomes (IRTPRO) v2.1 [40] was used for the IRT analysis, and the SAS 9.1 software [41] was used for the remaining analyses.

## Results

### Description of sample

Table 2 shows the characteristics of 469 parents of children aged 8–16 years. The average ages were 11.96 years old (SD = 2.55) for the children and 41.46 years old (SD = 11.73) for the parents. A plurality of the parents were white (49.7%), and the majority had received at least a high school diploma or equivalent degree (75.5%). A third (33.3%) of the families had an income of $20,000 or above.

**Table 2:** Demographic characteristics (N = 469).

Characteristics	N (%) or mean (SD)
Child age, years	11.96 (2.55)
Parent age, years	41.46 (11.73)
Child gender, %	
Male	221 (47.1)
Female	248 (52.9)
Child race/ethnicity, %	
White	208 (44.3)
Black	143 (30.5)
Hispanic	53 (11.3)
Other	62 (13.2)
Refused	3 (0.6)
Parent race/ethnicity, %	
White	233 (49.7)
Black	140 (29.9)
Hispanic	51 (10.9)
Other	43 (9.2)
Refused	2 (0.4)
Parent education, %	
Less than HS	112 (23.9)
GED/HS degree	183 (39.0)
Vocational/some college/AA Degree	133 (28.4)
College graduate	30 (6.4)
Graduate degree	8 (1.7)
Marital status, %	
Married	187 (39.9)
Single	134 (28.6)
Other	143 (30.49)
Family income, %	
<$9,999	132 (28.2)
$10,000–$19,999	153 (32.6)
$20,000+	156 (33.3)
Refused	28 (6.0)

SD: standard deviation.

#### Step 1: Mapping items from the three pediatric HRQoL instruments using the ICF-CY framework

Table 3 displays an overview regarding the mapping of the concepts of specific items to the second-level categories of the ICF-CY. The concepts represented by the items were linked to 24 different second-level ICF-CY categories, including nine categories in the body functions component, 13 categories in the activities and participation component, and two categories in the environmental factor component. None of the items from the three HRQoL instruments were assigned to the body structures component, and three items each were not definable or not covered.

**Table 3:** Specific items and their linkage to ICF-CY second-level categories for the development of item banks.

Domain	Item	Item question	ICF-CY category
Personality	Kindl11	My child felt pleased with him-/herself	b126 Temperament and personality functions
	Kindl20	My child felt different from other children	b126 Temperament and personality functions
	Kdscrn10	Has your child felt cheerful?	b126 Temperament and personality functions
	Kdscrn12	Has your child felt that he/she does everything badly?	b126 Temperament and personality functions
	Kdscrn19	Has your child been happy with the way he/she is?	b126 Temperament and personality functions
	Kdscrn43	Has your child been able to rely on his/her friends?	b126 Temperament and personality functions
Emotional function	Pedsql13	Worrying about what will happen to him/her	b152 Emotional functions
	Kindl5	My child had fun and laughed a lot	b152 Emotional functions
	Kindl8	My child felt scared or unsure of her/him-self	b152 Emotional functions
	Kdscrn6	Has your child felt that life was enjoyable?	b152 Emotional functions
	Kdscrn13	Has your child felt sad?	b152 Emotional functions
	Kdscrn14	Has your child felt so bad that he/she didn't want to do anything?	b152 Emotional functions
	Kdscrn15	Has your child felt that everything in his/her life goes wrong?	b152 Emotional functions
	Kdscrn16	Has your child felt fed up?	b152 Emotional functions
	Kdscrn17	Has your child felt lonely?	b152 Emotional functions
	Kdscrn18	Has your child felt under pressure?	b152 Emotional functions
	Kdscrn20	Has your child been happy with his/her clothes?	b152 Emotional functions
	Kdscrn31	Has your child been happy at home?	b152 Emotional functions
	Kdscrn44	Has your child been happy at school?	b152 Emotional functions
	Kdscrn50	Has your child been afraid of other girls and boys?	b152 Emotional functions
Mobility	Pedsql4	Lifting something heavy?	d430 Lifting and carrying objects
	Pedsql1	Walking more than one block?	d450 Walking
	Kdscrn2	Has your child felt physically well and fit?	d455 Moving around
	Pedsql2	Running?	d455 Moving around
Energy	Pedsql8	Low energy level?	b130 Energy and drive functions
	Kindl3	My child was tired and worn-out	b130 Energy and drive functions
	Kindl6	My child didn't feel much like doing anything	b130 Energy and drive functions
	Kdscrn5	Has your child felt full of energy?	b130 Energy and drive functions
	Pedsql12	Trouble sleeping?	b134 Sleep functions
Social function	Kindl19	My child got along well with his/her friends	d750 Informal social relationships
	Kdscrn27	Has your child had enough time to meet friends?	d750 Informal social relationships
	Kdscrn38	Has your child spent time with his/her friends?	d750 Informal social relationships
	Kdscrn40	Has your child had fun with his/her friends?	d750 Informal social relationships
	Kdscrn41	Has your child and his/her friends helped each other?	d750 Informal social relationships
	Kdscrn51	Have other girls and boys made fun of your child?	d750 Informal social relationships
Task accomplishment	Pedsql22	Missing school because of not feeling well?	d230 Carrying out daily routine
	Pedsql5	Taking a bath or shower by him or herself?	d510 Washing oneself
	Pedsql6	Doing chores around the house?	d640 Doing housework
	Pedsql3	Participating in sports activity or exercise?	d920 Recreation and leisure
Family function	Kindl13	My child got on well with us as parents	d760 Family relationships
	Kdscrn29	Has your child felt understood by his/her parents?	d760 Family relationships
	Kdscrn30	Has your child felt loved by his/her friends?	d760 Family relationships
	Kdscrn33	Has your child felt that his/her parent(s) treated him/her fairly?	d760 Family relationships
	Kdscrn34	Has your child been able to talk to his/her parent(s) when he/she wanted to?	d760 Family relationships
School function	Pedsql21	Keeping up with schoolwork?	d820 School education
	Kindl22	My child enjoyed the school lessons	d820 School education
	Kdscrn45	Has your child gotten on well at school?	d820 School education
	Kdscrn49	Has your child gotten along well with his/her teachers?	d820 School education
	Kindl17	My child played with friends	d880 Engagement in play
	Pedsql17	Not being able to do things that other children/teens his or her age can do?	nd
	Pedsql18a	Keeping up with other teens	nd
	Kindl23	My child worried about his/her future	nd
	Kindl1	My child felt ill	nc
	Kindl14	My child felt fine at home	nc
	Kdscrn1	In general, how would your child rate his/her health?	nc

Kdscrn: KIDSCREEN-52; Kindl: KINDL-R; Pedsql: PedsQL; nd: not definable; nc: not covered.

All Kindl items start with “During the past week….”; All Kdscrn items start with “Please try to remember your child's experiences over the last month…”; and all Pedsql items start with “How much of a problem has your child/teen had with…”.

Meaningful concepts in items from the KIDSCREEN-52, KINDL-R, and PedsQL were almost equally represented by the body functions categories: six, four, and five second-level categories, respectively. In contrast, meaningful concepts of items from the PedsQL were greatly represented by the activities and participation categories: 10 second-level categories for the PedsQL compared with six and five second-level categories for KINDL-R and KIDSCREEN-52, respectively. The environmental factor component was not well represented by any of the three instruments as only one item each from the KIDSCREEN-52 and PedsQL was linked to a single category of the environmental factor component.

Appendix S1 shows the detailed mapping results between the concepts of individual items from the three instruments and the specific codes for the ICF-CY's body functions, activity and participation, and environmental factor components. This specifically informs how each item is represented by the ICF-CY components and categories. A higher representation for the items from the HRQoL instrument to a specific ICF-CY category suggests that the same concept was captured by potentially redundant items from this specific instrument. Items from the three instruments that were linked to the same ICF-CY category represented the same underlying construct, and a specific domain comprising items measuring this construct was created. As a result, 10 initial HRQoL domains were created based on the criterion of all the items in that domain that potentially measured the same underlying construct and had a minimum of four items per domain, including personality, emotion, mobility, energy, social function, task accomplishment, family function, school function, cognition, and experience of self (Table 3 and Appendix S2). However, two of the ten domains (cognition and experience of self) were not considered further as they contained only a few items measuring those domains. Three of the six items measuring the experience of self domain were deleted because they had factor loadings <0.4 (see Step 2 below and Appendix S2). The remaining three items were considered too few to measure the experience of self. Four out of the five items from the cognition domain were assigned to different ICF-CY categories (b140, b144, b160, and b164), suggesting that these items might not measure the same underlying construct (Appendix S2).

#### Step 2: Psychometric analysis using IRT

CFA was performed to test the structural validity of the individual HRQoL domains with the goal of retaining items to meet the assumptions of unidimensionality and local independence to conduct the IRT analysis (Table 3 and Appendix S2). In the CFA, items that were significantly associated with the corresponding domains with factor loadings (λ>0.4; *p*<0.05) and residual covariance (<10.0) were considered for retention (Table 3). As a result, a total of six items were deleted due to either a low factor loading (<0.4) or a high residual covariance (>10.0), including one item in the mobility domain, three items in the social function domain, and two items in the task accomplishment domain (Appendix S2).

Item parameter estimates from the GRM for eight specific HRQoL domains are presented in Table 4. Items that did not satisfy one of the criteria explained in the Methods section were excluded from the analysis (Appendix S2). For example, item 23 of the PedsQL (name: Pedsql23) was deleted because of a poor item fit and a high correlation (>10.0) with item 22 from the PedsQL (name: Pedsql22). A total of 21 items were deleted across different domains due to poor item fit, high correlation, and/or poor discrimination value (Appendix S2). Overall, we did not consider 50 items to be assigned to any of the eight domains, leaving 49 items in the eight item banks that measure eight HRQoL domains (Table 3, Table 4, and Appendix S2). After deleting specific items across different domains, the RMSEA values for all eight domains suggested a good model fit (<0.08), and the marginal reliability of the eight domains ranged from 0.63–0.87. The majority of the threshold parameters (representing item difficulty) corresponding to the response categories of each item in a specific domain had negative values, suggesting that the majority of items captured lower levels of underlying HRQoL (Table 4).

**Table 4:** Item parameter estimates using item response theory for individual HRQoL domains.

Domain	Item	Discrimination parameter	Location parameters (standard error)				Chi-square	Probability
		a (se)	b_1_ (se)	b_2_ (se)	b_3_ (se)	b_4_ (se)		
Personality trait	Kindl11	1.72 (0.19)	−2.7 (0.25)	−2.22 (0.19)	−0.97 (0.1)	0.17 (0.08)	40.34	0.21
	Kindl20	1.28 (0.16)	−3.0 (0.34)	−2.27 (0.24)	−0.81 (0.11)	−0.32 (0.09)	46.35	0.20
	Kdscrn10	1.83 (0.20)	−3.79 (0.49)	−2.98 (0.29)	−1.1 (0.1)	0.44 (0.08)	44.09	0.02
	Kdscrn12	1.35 (0.16)	−3.3 (0.36)	−2.7 (0.27)	−0.86 (0.11)	0.05 (0.09)	58.39	0.01
	Kdscrn19	2.23 (0.26)	−2.61 (0.23)	−2.36 (0.19)	−1.11 (0.09)	0.01 (0.07)	37.92	0.12
	Kdsrcn43	0.82 (0.12)	−3.82 (0.55)	−2.95 (0.42)	0.23 (0.13)	1.90 (0.28)	52.01	0.06
	M_2_ = 978.80, df (234), *p* = 0.0001, RMSEA = 0.08; Marginal reliability: 0.76							
Emotion	Pedsql13	1.45 (0.15)	−3.06 (0.30)	−2.57 (0.24)	−1.00 (0.11)	−0.33 (0.09)	84.59	0.05
	Kindl5	1.51 (0.15)	−3.45 (0.36)	−2.73 (0.25)	−1.26 (0.12)	−0.20 (0.08)	101.17	0.0007
	Kindl8	1.59 (0.19)	−4.15 (0.57)	−2.82 (0.28)	−1.66 (0.15)	−0.96 (0.10)	58.54	0.10
	Kdscrn6	1.91 (0.17)	−2.58 (0.21)	−1.97 (0.15)	−1.09 (0.09)	0.24 (0.08)	72.26	0.20
	Kdscrn13	1.56 (0.15)	−3.95 (0.47)	−2.95 (0.27)	0.02 (0.08)	1.06 (0.12)	52.32	0.24
	Kdscrn14	2.06 (0.20)	−2.95 (0.27)	−2.56 (0.21)	−0.76 (0.08)	−0.14 (0.07)	59.13	0.18
	Kdscrn15	2.15 (0.20)	−2.66 (0.22)	−2.20 (0.16)	−0.88 (0.08)	−0.26 (0.07)	49.54	0.65
	Kdscrn16	2.07 (0.19)	−2.45 (0.19)	−2.12 (0.16)	−0.68 (0.08)	−0.06 (0.07)	54.66	0.53
	Kdscrn17	2.55 (0.26)	−2.48 (0.19)	−2.26 (0.16)	−0.74 (0.07)	−0.20 (0.07)	63.67	0.05
	Kdscrn18	1.82 (0.17)	−3.19 (0.31)	−2.34 (0.19)	−0.37 (0.08)	0.16 (0.08)	60.68	0.28
	Kdscrn20	1.13 (0.13)	−4.05 (0.49)	−3.34 (0.37)	−1.55 (0.17)	−0.28 (0.10)	79.29	0.09
	Kdscrn31	1.58 (0.16)	−4.17 (0.55)	−3.44 (0.36)	−1.80 (0.15)	−0.19 (0.08)	62.62	0.05
	Kdscrn44	1.28 (0.13)	−3.07 (0.30)	−2.01 (0.19)	−0.70 (0.10)	0.79 (0.12)	107.46	0.007
	Kdscrn50	1.10 (0.14)	−4.29 (0.55)	−3.63 (0.43)	−1.22 (0.15)	−0.53 (0.11)	68.40	0.17
	M_2_ = 3926.04, df (1442), *p* = 0.0001, RMSEA = 0.06; Marginal reliability: 0.87							
Mobility	Pedsql4	1.98 (0.25)	−2.21 (0.20)	−1.99 (0.17)	−0.92 (0.09)	−0.56 (0.08)	53.36	0.008
	Pedsql1	3.35 (0.59)	−2.06 (0.16)	−1.58 (0.12)	−1.17 (0.09)	−0.87 (0.07)	40.03	0.13
	Kdscrn2	0.82 (0.13)	−3.09 (0.45)	−2.41 (0.35)	−1.07 (0.19)	0.97 (0.19)	34.25	0.51
	Pedsql2	3.70 (0.64)	−1.70 (0.13)	−1.48 (0.11)	−0.96 (0.08)	−0.62 (0.07)	42.39	0.02
	M_2_ = 351.01, df (92), *p* = 0.0001, RMSEA = 0.08; Marginal reliability: 0.63							
Energy	Pedsql8	2.53 (0.45)	−2.46 (0.24)	−1.97 (0.18)	−0.76 (0.09)	−0.28 (0.07)	36.96	0.18
	Kindl3	1.31 (0.18)	−3.60 (0.45)	−2.83 (0.32)	−1.03 (0.13)	−0.29 (0.09)	35.89	0.25
	Kindl6	1.39 (0.19)	−3.19 (0.36)	−2.52 (0.27)	−0.98 (0.12)	−0.19 (0.09)	66.14	0.002
	Kdscrn5	1.24 (0.16)	−3.84 (0.48)	−3.18 (0.37)	−1.42 (0.17)	0.09 (0.09)	38.88	0.13
	Pedsql12	1.26 (0.17)	−3.40 (0.41)	−2.62 (0.30)	−1.28 (0.16)	−0.62 (0.11)	46.59	0.06
	M_2_ = 497.42, df (155), *p* = 0.0001, RMSEA = 0.07; Marginal reliability: 0.69							
Social function	Kindl19	1.46 (0.16)	−3.45 (0.36)	−2.85 (0.27)	−1.26 (0.13)	−0.00 (0.09)	48.23	0.03
	Kdscrn27	1.54 (0.18)	−4.30 (0.58)	−3.83 (0.45)	−1.46 (0.14)	−0.48 (0.09)	37.05	0.06
	Kdscrn38	1.58 (0.16)	−3.17 (0.30)	−2.54 (0.22)	−0.42 (0.09)	0.72 (0.10)	48.05	0.03
	Kdscrn40	3.36 (0.43)	−2.91 (0.26)	−2.56 (0.20)	−1.06 (0.08)	−0.23 (0.06)	49.94	0.0004
	Kdscrn41	2.84 (0.33)	−2.83 (0.24)	−2.47 (0.19)	−0.60 (0.07)	0.08 (0.07)	35.30	0.05
	Kdscrn51	0.90 (0.12)	−4.19 (0.56)	−3.06 (0.39)	−0.23 (0.12)	0.49 (0.13)	76.11	0.0002
	M_2_ = 833.87, df (234), *p* = 0.0001, RMSEA = 0.07; Marginal reliability: 0.79							
Task accomplishment	Pedsql22	0.74 (0.13)	−5.19 (0.90)	−4.19 (0.71)	−1.30 (0.24)	0.19 (0.15)	55.68	0.006
	Pedsql5	3.86 (1.22)	−1.40 (0.13)	−1.29 (0.12)	−1.02 (0.10)	−0.94 (0.09)	29.16	0.21
	Pedsql6	2.25 (0.32)	−1.68 (0.14)	−1.50 (0.13)	−0.45 (0.08)	−0.14 (0.07)	28.20	0.51
	Pedsql3	1.49 (0.21)	−2.32 (0.25)	−2.06 (0.22)	−1.21 (0.14)	−0.67 (0.10)	44.38	0.13
	M_2_ = 235.41, df (92), *p* = 0.0001, RMSEA = 0.06; Marginal reliability: 0.64							
Family function	Kindl13	1.15 (0.15)	−3.31 (0.40)	−2.78 (0.32)	−1.45 (0.18)	−0.01 (0.10)	73.88	0.0001
	Kdscrn29	1.97 (0.22)	−2.27 (0.19)	−1.69 (0.14)	−0.65 (0.08)	0.53 (0.09)	99.67	0.0001
	Kdscrn30	2.59 (0.39)	−2.66 (0.25)	−2.15 (0.18)	−1.61 (0.13)	−0.64 (0.07)	67.24	0.0001
	Kdscrn33	1.48 (0.17)	−2.55 (0.25)	−2.27 (0.22)	−0.92 (0.11)	0.26 (0.09)	43.82	0.06
	Kdscrn34	1.61 (0.23)	−3.29 (0.39)	−3.11 (0.35)	−1.87 (0.19)	−0.92 (0.10)	29.10	0.26
	M_2_ = 492.80, df (155), *p* = 0.0001, RMSEA = 0.07; Marginal reliability: 0.73							
School function	Pedsql21	0.98 (0.13)	−2.18 (0.27)	−1.87 (0.23)	0.00 (0.11)	0.67 (0.14)	79.56	0.0001
	Kindl22	1.91 (0.22)	−2.05 (0.18)	−1.47 (0.13)	−0.31 (0.08)	0.52 (0.09)	61.34	0.004
	Kdscrn45	2.02 (0.25)	−2.44 (0.22)	−1.57 (0.13)	−0.70 (0.08)	0.36 (0.08)	50.90	0.03
	Kdscrn49	2.00 (0.25)	−2.74 (0.26)	−2.41 (0.22)	−1.05 (0.11)	−0.02 (0.07)	49.73	0.005
	Kindl17	0.57 (0.11)	−5.69 (1.10)	−4.23 (0.80)	−0.98 (0.24)	1.46 (0.32)	41.84	0.052
	M_2_ = 610.77, df (155), *p* = 0.0001, RMSEA = 0.08; Marginal reliability: 0.74							

RMSEA: root mean square error of approximation.

#### Step 3: Validation analysis using known-groups approach

Table 5 shows known-groups validity related to CSHCN for the eight specific domains. Overall, the eight domains were able to distinguish the underlying HRQoL between children with and without special health care needs. Bivariate analyses suggested that the HRQoL domain scores for seven domains (with the exception of personality) in the CSHCN were significantly lower than those in the children without special health care needs (p<0.05). The magnitude of ES was larger for domains of emotional function (ES = 0.65), energy (ES = 0.65), and school function (ES = 0.63) compared with the other domains (ES = 0.32 to 0.58; excluding the personality domain). Similar findings were replicated after adjusting for covariates (children's age and gender and parent's race and education).

**Table 5:** Known-groups validity for individual domain scores among children with special health care needs (CSCHN) compared with children without special health care needs.

Domains	CSCHN	Children without special health care needs	Difference (effect size)	Difference (effect size)
			Bivariate analysis	Multivariate analysis#
Personality	0.069 (0.879)	−0.049 (0.862)	−0.118 (−0.137)	−0.124 (0.142)
Emotional function	−0.337 (0.942)	0.226 (0.863)	0.562 (0.651)***	0.570 (0.609)***
Mobility	−0.245 (0.892)	0.167 (0.714)	0.415 (0.581)***	0.472 (0.581)***
Energy	−0.282 (0.901)	0.189 (0.726)	0.471 (0.649)***	0.488 (0.587)***
Social function	−0.213 (0.901)	0.143 (0.863)	0.356 (0.413)***	0.361 (0.402)***
Task accomplishment	−0.202 (0.780)	0.136 (0.804)	0.338 (0.423)***	0.379 (0.466)***
Family function	−0.157 (0.860)	0.106 (0.834)	0.263 (0.315)**	0.216 (0.255)**
School function	−0.293 (0.882)	0.196 (0.774)	0.489 (0.632)***	0.499 (0.587)***

** *p*<0.01.

*** *p*<0.001.

# Controlling for child's age and gender and parent's race and education.

## Discussion

This study shows that the contents of items from the three pediatric HRQoL legacy instruments were well represented by the ICF-CY categories. Specifically, compared with the KIDSCREEN-52 and KINDL, the PedsQL was found to better represent the activity and participation components of the ICF-CY. The KINDL-R and PedsQL corresponded less with respect to the environmental factors, which is consistent with a previous study that linked items of these instruments to the ICF-CY categories [5]. However, we only linked one item in the KIDSCREEN-52 to a single category of the environmental factor component, which contrasts with the results of a previous study showing that 24% of the items in the KIDSCREEN-52 were able to link to six different categories in the environmental factor component [5]. This discrepancy might have arisen because the latter study linked each item to more than one concept, resulting in the total number of concepts exceeding the number of items in the instrument. However, because our purpose was to develop different item banks with each capturing a unidimensional concept of HRQoL, we chose the most relevant ICF-CY category to represent the content of an individual item. Since our selection criteria for linkage methodology was different from the previous studies [4], [5], [22]–[24], our findings do not allow for recommending which pediatric HRQOL instrument should be chosen. Instead, we argue that different pediatric HRQOL instruments contain items of different measurement properties and the inclusion of various items from different instruments will strengthen item banks with robust measurement properties.

This study uses the rules recommended by Cieza et al. [18] to link items from the three pediatric HRQoL instruments to the specific ICF-CY categories. Consequently, eight specific unidimensional domains emerged, which provides a foundation for developing item banks to measure pediatric HRQoL. CFA were used to test for structural validity, and IRT was used to calibrate items from different instruments on the same metric and to calculate domain scores for individuals. Items were deleted from the item banks if they were not represented by the appropriate ICF-CY categories or if they demonstrated poor performance on the basis of psychometric properties. This combined use of qualitative and quantitative approaches allowed for the concomitant establishment of the content validity of individual domains and the confirmation of content validity using IRT. This strategy directly led to robust item banks that measure unidimensional HRQoL constructs. We identified several items represented by the same ICF-CY category, indicating an overlap in the concepts across different pediatric HRQoL instruments. Our linkage also identified some items that were not included in the item banks as the ICF-CY unfortunately does not represent meaningful concepts for these items. Future studies might replicate our approach to explore the potential similarities or discrepancies in these findings and expand our item banks based on the ICF-CY framework to link existing items from other pediatric HRQoL instruments or to add novel items.

The linking process was conducted based on a parent-proxy report rather than on a child self-report. The use of a parent versus a child version of the instruments might not result in discrepant item mapping results because the contents in the child and parent versions are almost the same. However, data collected from a parent-proxy versus a child self-report might lead to different quantitative results in terms of construct validity and item parameters because parents and children possess different perceptions with regard to interpreting and answering pediatric HRQoL items [42]. A greater discrepancy between proxy- and self-reports has been found in the more abstract domains (e.g., emotional functioning) compared with the less abstract domains (e.g., physical functioning) [43].

We found that the mean underlying HRQoL scores for seven of the eight domains (except personality) were significantly lower (*p*<0.05) in the CSHCN compared to the children without special health care needs in the bivariate and multivariate analyses (Table 5). These findings suggest a high level capacity to discriminate and differentiate CSHCN with respect to different emotional (emotional function and social function) and activity and participation (mobility, energy, task accomplishment, family function, and school function) domains compared with children without special health care needs. The ES for the underlying HRQoL scores was the highest for the emotional function domain, followed by the energy domain. This finding echoes our previous study suggesting that CSHCN require more physical, developmental, and emotional support than children without special health care needs [44]. Not surprisingly, children with and without special health care needs had similar personality domain scores because personality is a trait that is less likely to be related to different levels of special health care needs.

The linkage of ICF-CY categories to the concepts measured by the items in the three pediatric HRQoL instruments helped with the development of the item banks, which in turn assists clinicians in using the measurement tools to evaluate the comparative effectiveness of different interventions [3], [45]. However, there is limited evidence of the ability of HRQoL tools to measure the impact of environmental and personal factors on a child's health status. The ICF-CY framework provides an important foundation for creating the unidimensional item banks. Through the ICF-CY framework we were able to identify items from different pediatric HRQoL instruments that specifically capture life experiences, activities and participation appropriate for the child's age and developmental stage. The broader perspective embedded in the ICF-CY framework provides a valuable opportunity to develop measures to capture factors influencing physical and emotional functioning and activity and participation, further facilitating the ability of researchers and clinicians to design appropriate interventions targeting these modifiable factors [3].

The ICF framework was primarily developed to measure disability, functional status, and social participation rather than quality of life [46]. The components of the ICF are more objective (e.g., ability to perform specific functioning) than subjective (e.g., satisfaction with health). In this regard, the linkage exercise using the ICF-CY framework might not have distinguishable concepts for some items that measure specific pediatric HRQoL. The concepts for some categories in the ICF-CY are general and thus susceptible to a broader description of the meaningful concepts for the items. For example, the ICF-CY body function category b152 (emotional functions) can be explained by several aspects regarding emotions such as sadness, laughter, and fear. In turn, this general nature resulted in linking a specific ICF-CY category to several items from the pediatric HRQoL instruments.

Several limitations should be noted when interpreting the findings of this study. First, the generalizability of the findings is limited due to the use of a Medicaid population from Florida. Second, the linkage was conducted based on the perspective of the investigators and did not include the perspectives of the parents and children themselves. Indeed, investigators, parents and children may interpret the meaning of items differently [47]. With an emphasis on a patient-centeredness approach, future studies might consider engaging parents and children as stakeholders alongside researchers in the item mapping process to strengthen the content validity and develop a robust methodology for the synergy of findings from different stakeholders. Third, the CSHCN status reported by parents was used for evaluating the known-groups validity. This information may result in varying outcomes compared with the use of categorical approaches, such as disease diagnoses. Finally, the survey response rate (30.2%) was lower than that of previous studies (usually 60%) focusing on Medicaid pediatric populations [48]–[50]. However, responders and nonresponders did not differ significantly on children's age and sex (*p*>0.05). The lower response rate is in part due to the inclusion of a lengthy survey (approximately 50 minutes per survey) that precludes subjects from the study participation. Although the lower response rate may threaten the generalizability of our findings, this population is important to assess because they were below 100% of the federal poverty level and possess greater risk of poor health status due to poor socioeconomic circumstances.

## Conclusions

The ICF-CY serves as a useful framework to compare the concepts of items from the three pediatric HRQoL legacy instruments and to generate item banks for a pediatric population. This study provides useful insights regarding the content coverage of items from three instruments represented by the ICF-CY framework. This study has implications for researchers to refine pediatric HRQoL instruments and for clinicians to use these instruments in clinical practice.

## Supporting Information

Appendix S1 (DOCX)

Appendix S2 (DOCX)

## References

[1] World Health Organization (2001) International Classification of Functioning, Disability and Health: ICF. Geneva: WHO.

[2] World Health Organization. International classification of functioning, disability and health - version for children and youth (2007) Geneva: WHO Library Cataloguing-in-Publication Data.

[3] Fava L, Muehlan H, Bullinger M (2009) Linking the DISABKIDS modules for health-related quality of life assessment with the International Classification of Functioning, Disability and Health (ICF). Disabil Rehabil 31: 1943–54. 19479567 10.1080/09638280902874188

[4] Fayed N, Cieza A, Bickenbach J (2012) Illustrating child-specific linking issues using the Child Health Questionnaire. Am J Phys Med Rehabil 91(suppl): S189–S198. 22193318 10.1097/PHM.0b013e31823d53cf

[5] Petersson C, Simeonsson RJ, Enskar K, Huus K (2013) Comparing children's self-report instruments for health-related quality of life using the International Classification of Functioning, Disability and Health for Children and Youth (ICF-CY). Health Qual Life Outcomes 11: 75. 23642162 10.1186/1477-7525-11-75 PMC3648353

[6] Starfield B, Riley AW, Green BF, Ensminger ME, Ryan SA, et al (1995) The adolescent child health and illness profile: A population-based measure of health. Med Care 33: 553–566. 7739277 10.1097/00005650-199505000-00008

[7] Landgraf JM, Abetz L, Ware JE (1996) Child Health Questionnaire (CHQ): A user's manual. The Health Institute New England Medical: Boston, MA.

[8] Ravens-Sieberer U, Gosch A, Rajmil L, Erhart M, Bruil J, et al (2005) KIDSCREEN-52 quality-of-life measure for children and adolescents. Expert Rev Pharmacoecon Outcomes Res 5: 353–364. 19807604 10.1586/14737167.5.3.353

[9] Ravens-Sieberer U, Bullinger M (1998) Assessing health-related quality of life in chronically ill children with the german KINDL: First psychometric and content analytical results. Qual Life Res 7: 399–407. 9691720 10.1023/a:1008853819715

[10] Varni JW, Seid M, Rode CA (1999) The PedsQL™: Measurement model for the pediatric quality of life inventory. Med Care 37: 126–139. 10024117 10.1097/00005650-199902000-00003

[11] Varni JW, Burwinkle TM, Lane MM (2005) Health-related quality of life measurement in pediatric clinical practice: An appraisal and precept for future research and application. Health Qual Life Outcomes 3: 34. 15904527 10.1186/1477-7525-3-34 PMC1156928

[12] De Civita M, Regier D, Alamgir AH, Anis AH, Fitzgerald MJ, et al (2005) Evaluating health-related quality-of-life studies in paediatric populations: Some conceptual, methodological and developmental considerations and recent applications. Pharmacoeconomics 23: 659–685. 15987225 10.2165/00019053-200523070-00003

[13] Waters E, Davis E, Ronen GM, Rosenbaum P, Livingston M, et al (2009) Quality of life instruments for children and adolescents with neurodisabilities: How to choose the appropriate instrument. Dev Med Child Neurol 51: 660–669. 19627340 10.1111/j.1469-8749.2009.03324.x

[14] Kenzik KM, Huang I-C, Tuli SY, Nackashi JA, Revicki D, et al.. (2011) Head-to-head comparison of four legacy pediatric health-related quality of life instruments: a study on parent proxy-report. Qual Life Res (Suppl 1): 98.

[15] Kenzik KM, Tuli SY, Revicki D, Shenkman EA, Huang I-C (2014) Comparison of 4 pediatric health-related quality of life instruments: a study on a Medicaid population. Med Dec Making 34(5): 590–602. 10.1177/0272989X14529846 PMC4199940 24739533

[16] Hays RD, Morales LS, Reise SP (2000) Item response theory and health outcomes measurement in the 21st century. Med Care 38(9 Suppl): II28–42. 10.1097/00005650-200009002-00007 PMC1815384 10982088

[17] Hambleton RK, Jones RW (1993) Comparison of classical test theory and item response theory and their applications to test development. Educ Meas Issues Pract 12: 38–47.

[18] Cieza A, Geyh S, Chatterji S, Kostanjsek N, Ustun B, et al (2005) ICF linking rules: an update based on lessons learned. J Rehabil Med 37(4): 212–218. 16024476 10.1080/16501970510040263

[19] The World Health Organization's (WHO) International Classification of Functioning, Disability and Health, Children and Youth (ICF-CY). ICF-CY Developmental Code Sets (2014). Available: http://www.icf-cydevelopmentalcodesets.com/. Accessed 2014 April 8.

[20] Cieza A, Stucki G (2005) Content comparison of health-related quality of life (HRQOL) instruments based on the international classification of functioning, disability and health (ICF). Qual Life Res 14: 1225–1237. 16047499 10.1007/s11136-004-4773-0

[21] Geyh S, Cieza A, Kollerits B, Grimby G, Stucki G (2007) Content comparison of health-related quality of life measures used in stroke based on the international classification of functioning, disability and health (ICF): a systematic review. Qual Life Res 16: 833–851. 17294283 10.1007/s11136-007-9174-8

[22] Bendixen RM, Senesac C, Lott DJ, Vandenborne K (2012) Participation and quality of life in children with Duchenne muscular dystrophy using the International Classification of Functioning, Disability, and Health. Health Qual Life Outcomes 10: 43. 22545870 10.1186/1477-7525-10-43 PMC3358238

[23] Krasuska M, Riva S, Fava L, von Mackensen S, Bullinger M (2012) Linking quality-of-life measures using the International Classification of Functioning, Disability and Health and the International Classification of Functioning, Disability and Health-Children and Youth Version in chronic health conditions: the example of young people with hemophilia. Am J Phys Med Rehabil 91(13 Suppl 1)S74–S83. 22193314 10.1097/PHM.0b013e31823d4f35

[24] Riva S, Bullinger M, Amann E, von Mackensen S (2010) Content comparison of haemophilia specific patient-rated outcome measures with the International classification of functioning, disability and health (ICF, ICF-CY). Health Qual Life Outcomes 8: 139. 21108796 10.1186/1477-7525-8-139 PMC3022566

[25] Lee AM (2011) Using the ICF-CY to organize characteristics of children's functioning. Disabil Rehabil 33(7): 605–16. 20695793 10.3109/09638288.2010.505993

[26] Riley AW (2004) Evidence that school-age children can self-report on their health. Ambul Pediatr 4: 371–376. 15264962 10.1367/A03-178R.1

[27] U.S. Department of Health and Human Services FDA Center for Drug Evaluation and Research; U.S. Department of Health and Human Services FDA Center for Biologics Evaluation and Research; U.S. Department of Health and Human Services FDA Center for Devices and Radiological Health (2006) Guidance for industry: patient-reported outcome measures: use in medical product development to support labeling claims: draft guidance. Health Qual Life Outcomes 4: 79. 17034633 10.1186/1477-7525-4-79 PMC1629006

[28] Varni JW, Limbers CA, Burwinkle TM (2007) Parent proxy-report of their children's health-related quality of life: an analysis of 13,878 parents' reliability and validity across age subgroups using the PedsQL 4.0 Generic Core Scales. Health Qual Life Outcomes 5: 2. 17201923 10.1186/1477-7525-5-2 PMC1769359

[29] Ingerski LM, Janicke DM, Silverstein JH (2007) Brief report: quality of life in overweight youth-the role of multiple informants and perceived social support. J Pediatr Psychol 32: 869–874. 17488775 10.1093/jpepsy/jsm026

[30] Solans M, Pane S, Estrada M, Serra-Sutton V, Berra S, et al (2008) Health-related quality of life measurement in children and adolescents: A systematic review of generic and disease-specific instruments. Value Health 11: 742–764. 18179668 10.1111/j.1524-4733.2007.00293.x

[31] Todd J, Armon C, Griggs A, Poole S, Berman S (2006) Increased rates of morbidity, mortality, and charges for hospitalized children with public or no health insurance as compared with children with private insurance in Colorado and the United States. Pediatrics 118: 577–585. 16882810 10.1542/peds.2006-0162

[32] Bethell CD, Read D, Stein REK, Blumberg SJ, Wells N, et al (2002) Identifying children with special health care needs: Development and evaluation of a short screening instrument. Ambul Pediatr 2: 38–48. 11888437 10.1367/1539-4409(2002)002<0038:icwshc>2.0.co;2

[33] The KIDSCREEN Group Europe (2006) The KIDSCREEN questionnaires: Quality of life questionnaires for children and adolescents handbook. Pabst Science Publishers: Lengerich, Germany.

[34] Brown TA (2006) Confirmatory factor analysis for applied research. The Guilford Press: New York: London.

[35] Chen W-H, Thissen D (1997) Local dependence indices for item pairs using item response theory. J Educ Behav Stat 22: 265–289.

[36] Maydeu-Olivares A, Joe H (2006) Limited information goodness-of-fit testing in multidimensional contingency tables. Psychometrika 71: 713–732.

[37] Maydeu-Olivares A, Joe H (2005) Limited and full information estimation and testing in 2^nd^ contingency tables: A unified framework. J Am Stat Assoc 100: 1009–1020.

[38] Cohen J (1988) Statistical power analysis for the behavioral sciences. L. Erlbaum Associates: Hillsdale, NJ.

[39] Jöreskog K, Sörbom D (1993) LISREL 8. Lincolnwood, Il: Scientific Software International, Inc.

[40] Cai L, du Toit SHC, Thissen D (2011) IRTPRO: Computer software. Chicago: SSI International.

[41] SAS Institute (2004) SAS users guide, version 9.1. Cary, NC; SAS institute, Inc.

[42] Huang I-C, Shenkman EA, Leite W, Knapp CA, Thompson LA, et al (2009) Agreement was not found in adolescent's quality of life rated by parents and adolescents. J Clin Epidemiol 62 (3): 337–346. 10.1016/j.jclinepi.2008.06.012 PMC3812676 18834712

[43] Theunissen NC, Vogel TG, Koopman HM, Verrips GH, Zwinderman KA, et al (1998) The proxy problem: child report versus parent report in health related quality of life measure. Qual Life Res 7: 387–397. 9691719 10.1023/a:1008801802877

[44] Huang I-C, Leite WL, Shearer P, Seid M, Revicki DA, et al (2011) Differential item functioning in quality of life measure between children with and without special health-care needs. Value Health 14 (6): 872–83. 10.1016/j.jval.2011.03.004 PMC3173710 21914509

[45] Rosenbaum P, Stewart D (2004) The World Health Organization International Classification of Functioning, Disability, and Health: A model to guide clinical thinking, practice and research in the field of cerebral palsy. Seminars Ped Neurology 11: 5–10. 10.1016/j.spen.2004.01.002 15132248

[46] Valderas JM, Alonso J (2008) Patient reported outcome measures: a model-based classification system for research and clinical practice. Qual Life Res 17: 1125–1135. 18836850 10.1007/s11136-008-9396-4

[47] Davis E, Nicolas C, Waters E, Cook K, Gibbs L, et al (2007) Parent-proxy and child self-reported health-related quality of life: using qualitative methods to explain the discordance. Qual Life Res 16: 863–71. 17351822 10.1007/s11136-007-9187-3

[48] Huang IC, Shenkman EA, Leite W, Knapp CA, Thompson LA, et al (2009) Agreement was not found in adolescents' quality of life rated by parents and adolescents. J Clin Epidemiol 62(3): 337–46. 18834712 10.1016/j.jclinepi.2008.06.012 PMC3812676

[49] Huang IC, Thompson LA, Chi YY, Knapp CA, Revicki DA, et al (2009) The linkage between pediatric quality of life and health conditions: establishing clinically meaningful cutoff scores for the PedsQL. Value Health 12(5): 773–81. 19508660 10.1111/j.1524-4733.2008.00487.x PMC4299816

[50] Huang IC, Wen PS, Revicki DA, Shenkman EA (2011) Quality of Life Measurement for Children with Life-Threatening Conditions: Limitations and a New Framework. Child Indic Res 4(1): 145–160. 21760876 10.1007/s12187-010-9079-x PMC3133777

