# Supplementary material for: Developing Item Banks for Measuring Pediatric Generic Health-Related Quality of Life: An Application of the International Classification of Functioning, Disability and Health for Children and Youth and Item Response Theory
Source: PLoS One. 2014 Sep 30;9(9):e107771. doi: 10.1371/journal.pone.0107771 (PMC4182329; doi:10.1371/journal.pone.0107771)
Supplement: Appendix S1 — (DOCX) [file pone.0107771.s001.docx]

Appendix 1 (web-only material): Mapping results between the concepts of individual items of three HRQoL instruments and specific codes for the ICF-CY’s body functions, activity and participation, and environmental factor components

| ICF-CY component and category | KIDSCREEN-52 | KINDL-R | PedsQL |
| --- | --- | --- | --- |
| Body functions |  |  |  |
| b126 Temperament and personality functions | 5 | 4 |  |
| b130 Energy and drive functions | 1 | 3 | 1 |
| b134 Sleep functions |  |  | 1 |
| b140 Attention functions | 1 |  | 1 |
| b144 Memory functions, unspecified |  |  | 1 |
| b152 Emotional functions, other specified | 15 | 4 | 4 |
| b160 Thought functions, other specified |  | 1 |  |
| b164 Time management | 1 |  |  |
| b180 Experience of self and time functions | 3 |  |  |
| b280 Sensation of pain |  | 1 |  |
| b289 Sensation of pain, other specified and unspecified |  |  | 1 |
| Activity and participation |  |  |  |
| d230 Carrying out daily routine |  |  | 2 |
| d240 Handling stress and other psychological demands |  | 1 |  |
| d430 Lifting and carrying objects |  |  | 1 |
| d450 Walking |  |  | 1 |
| d455 Moving around | 3 |  | 1 |
| d510 Washing oneself |  |  | 1 |
| d640 Doing housework |  |  | 1 |
| d720 Complex interpersonal interactions |  | 1 | 1 |
| d750 Informal social relationships | 8 | 2 | 1 |
| d760 Family relationships | 5 | 2 |  |
| d820 School education | 2 | 2 | 1 |
| d880 Engagement in play |  | 1 |  |
| d920 Recreation and leisure | 1 |  | 1 |
| Environmental factors |  |  |  |
| e165 Assets | 3 |  |  |
| e420 Individual attitudes of friends |  |  | 1 |
| Not defined |  | 1 | 2 |
| Not covered | 1 | 2 |  |
