# Supplementary material for: Developing Item Banks for Measuring Pediatric Generic Health-Related Quality of Life: An Application of the International Classification of Functioning, Disability and Health for Children and Youth and Item Response Theory
Source: PLoS One. 2014 Sep 30;9(9):e107771. doi: 10.1371/journal.pone.0107771 (PMC4182329; doi:10.1371/journal.pone.0107771)
Supplement: Appendix S2 — (DOCX) [file pone.0107771.s002.docx]

Appendix 2 (web-only material): Evidence for exclusion of specific items from item banks

| Domain | Item | Item question | ICF-CY category | Reason for exclusion |
| --- | --- | --- | --- | --- |
| Personality | Kindl9 | my child was proud of him-/herself | b126 Temperament and personality functions | Deleted^c^ – poor item fit and highly correlated (>10.0) with other items in this domain (kindl10 and kindl11) |
|  | Kindl10 | my child felt on top of the world | b126 Temperament and personality functions | Deleted^c^ – uses unusual term and highly correlated (>10.0) with items kindl9 and kindl11 |
|  | Kdscrn9 | Has your child been in a good mood? | b126 Temperament and personality functions | Deleted^c^ – highly correlated (>10.0) and shares the same content with several items in this domain (kdscrn10, kdscrn12, and kdscrn19) |
| Emotional function | Pedsql9 | feeling afraid or scared | b152 Emotional functions | Deleted^c^ – poor item fit and highly correlated (>10.0) with several items in this domain (pedsql10, pedsql11, pedsql13, and kindl8) |
|  | Pedsql10 | feeling sad or blue | b152 Emotional functions | Deleted^c^ – poor item fit and highly correlated (>10.0) with several items in this domain (pedsql11 and pedsql13) and shares the same content with kdscrn13 (although not locally dependent) |
|  | Pedsql11 | feeling angry | b152 Emotional functions | Deleted^c^ – highly correlated with pedsql13 (>10.0) |
|  | Kindl7 | my child felt alone | b152 Emotional functions | Deleted^c^ – poor item fit and highly correlated with kindl8 in this domain (>10.0) |
|  | Kindl24 | my child was afraid of bad marks or grades | b152 Emotional functions | Deleted^c^ – lower discrimination value (<1.0) |
|  | Kdscrn7 | Has your child felt pleased that he/she is alive? | b152 Emotional functions | Deleted^c^ – poor item fit and highly correlated (>10.0) with items kdscrn6 and kdscrn8 and uses an unusual term |
|  | Kdscrn8 | Has your child felt satisfied with his/her life? | b152 Emotional functions | Deleted^c^ – highly correlated (>10.0) with items kdscrn6 and kdscrn7 |
|  | Kdscrn46 | Has your child been satisfied with his/her teachers? | b152 Emotional functions | Deleted^c^ – highly correlated (>10.0) with item kdscrn44 |
|  | Kdscrn48 | Has your child enjoyed going to school? | b152 Emotional functions | Deleted^c^ – poor item fit and highly correlated (>10.0) with items kdscrn44 and kdscrn46 |
| Cognition | Pedsql19 | paying attention in class? | b140 Attention functions | Deleted^b^ – did not consider this item bank |
|  | Kdscrn47 | Has your child been able to pay attention? | b140 Attention functions | Deleted^b^ – did not consider this item bank |
|  | Pedsql20 | forgetting things? | b144 Memory functions | Deleted^b^ – did not consider this item bank |
|  | Kindl12 | my child had lots of good ideas | b160 Thought functions | Deleted^b^ – did not consider this item bank |
|  | Kdscrn24 | Has your child had enough time for him/herself? | b164 Higher-level cognitive functions | Deleted^b^ – did not consider this item bank |
| Mobility | Kdscrn3 | Has your child been physically active (e.g., running, climbing, biking)? | d455 Moving around | Deleted^a^ – highly correlated with kdscrn4 |
|  | Kdscrn4 | Has your child been able to run well? | d455 Moving around | Deleted^c^ – poor item fit and highly correlated (>10.0) with kdscrn2 and shares the same content with pedsql2 (although not locally dependent) |
| Energy | Kindl4 | my child felt strong and full of energy | b130 Energy and drive functions | Deleted^c^ – highly correlated (>10.0) with kindl2 and shares the same content with kdscrn5 (although not locally dependent) |
| Social function | Pedsql15 | other children/teens not wanting to be his/her friend? | d720 Complex interpersonal interactions | Deleted^a^ – highly correlated with pedsql_14 and kdscrn52 (>25.0) |
|  | Kindl15 | we quarreled at home | d720 Complex interpersonal interactions | Deleted^a^ – low factor loading (<0.40) |
|  | Pedsql14 | getting along with other children/teens? | d750 Informal social relationships | Deleted^a^ – low factor loading (<0.40) |
|  | Kindl18 | my child was liked by other kids | d750 Informal social relationships | Deleted^c^ – poor item fit and highly correlated (>10.0) with kindl19 |
|  | Kdscrn39 | has your child done things with other girls and boys? | d750 Informal social relationships | Deleted^c^ – poor item fit and highly correlated (>10.0) with items kdscrn40 and kdscrn42 |
|  | Kdscrn42 | Has your child been able to talk about everything with his/her friends? | d750 Informal social relationships | Deleted^c^ – poor item fit and highly correlated (>10.0) with kdscrn41 |
|  | Kdscrn52 | Have other girls and boys bullied your child? | d750 Informal social relationships | Deleted^c^ – poor discrimination value (<1.00) |
| Task accomplishment | Pedsql23 | missing school to go to doctor or hospital? | d230 Carrying out daily routine | Deleted^c^ – poor item fit and highly correlated (>10.0) with pedsql22 |
|  | Kindl21 | my child easily coped with schoolwork | d240 Handling stress and other psychological demands | Deleted^a^ – low factor loading (<0.40) |
|  | Kdscrn11 | Has your child had fun? | d920 Recreation and leisure | Deleted^a^ – low factor loading (<0.40) |
| Family function | Kindl16 | my child felt that I was bossing him/her around | d760 Family relationships | Deleted^c^ – highly correlated (>10.0) with kindl13 |
|  | Kdscrn32 | has your child felt that his/her parents had enough time for him/her? | d760 Family relationships | Deleted^c^ – highly correlated (>10.0) with kdscrn33 |
| Experience of self | Kdscrn21 | Has your child been worried about the way he/she looks? | b180 Experience of self and time functions | Deleted^b^ – did not consider this item bank |
|  | Kdscrn22 | Has your child felt jealous of the way other girls and boys look? | b180 Experience of self and time functions | Deleted^b^ – did not consider this item bank |
|  | Kdscrn23 | has your child wanted to change something about his/her body? | b180 Experience of self and time functions | Deleted^b^ – did not consider this item bank |
|  | Kdscrn25 | Has your child been able to do the things that he/she wants to do in his/her free time? | b180 Experience of self and time functions | Deleted^a^ – low factor loading (<0.40) |
|  | Kdscrn26 | Has your child had enough opportunity to be outside? | b180 Experience of self and time functions | Deleted^a^ – low factor loading (<0.40) |
|  | Kdscrn28 | Has your child been able to choose what to do in his/her free time? | b180 Experience of self and time functions | Deleted^a^ – low factor loading (<0.40) |
|  |  | Not sure how to include the items stated below |  |  |

Kdscm: KIDSCREEN-52; Kindl: KINDL-R; Pedsql: PedsQL; nd: not definable; nc: not covered

All kindl items start with “During the past week….”; kdscrn items start with “Please try to remember your child’s experiences over the last month…”; pedsql items start with “How much of a problem has your child/teen had with…”

a After mapping items from three pediatric HRQoL instruments using the ICF-CY framework, the first step was to conduct confirmatory factor analysis. These items were deleted when factor loadings were <0.4 or residual covariance was >0.1.

b These items were deleted since only a few items were measuring specific underlying constructs.

c These items were deleted when they did not satisfy specific criteria using graded response model explained in the methods section.

Kdscrn35, kdscrn36, kdscrn37, and pedsql16 were not considered since these items were found to represent environmental factors component

Kindl12 and pedsql17 were deleted since specific domains relevant to these items were not identified based on linking process
